# The moderating role of personality traits in the relationship between work and salivary cortisol: a cross-sectional study of 401 employees in 34 Canadian companies

**DOI:** 10.1186/s40359-015-0102-3

**Published:** 2015-12-14

**Authors:** Annick Parent-Lamarche, Alain Marchand

**Affiliations:** School of Industrial Relations, University of Montreal, C.P. 6128, Succ. Centre-ville, Montreal, QC H3C 3 J7 Canada

**Keywords:** Salivary cortisol, Work conditions, Personality traits, Big Five, Self-esteem

## Abstract

**Background:**

The objective of this study was to evaluate the contribution of personality traits in explaining the relationship between workplace stressors and variations in salivary cortisol concentrations.

**Method:**

Multilevel regression analyses were performed on a sample of 401 employees from 34 Quebec firms. Saliva samples were collected five times a day (on awakening, 30 min after awakening, and at 2 p.m., 4 p.m., and bedtime). Sample collection was repeated on three days (1 rest day, 2 working days). Work-related variables comprised skill utilization, decision authority, psychological demands, physical demands, job insecurity, irregular schedule, number of working hours, and social support from coworkers and supervisors. Personality traits comprised self-esteem, locus of control, and the Big Five.

**Results:**

Cortisol levels at awakening and 30 min later were significantly higher for work days than for days off. Psychological demands and job insecurity were associated with lower cortisol levels at bedtime. Also, self-esteem moderated the relationship between physical demands and cortisol levels at awakening and 4 p.m. Agreeableness was associated with lower cortisol levels at awakening and at 2 p.m. and further moderated the relationship between number of hours worked and cortisol at 2 p.m. Neuroticism moderated the relationship between coworker support and cortisol at bedtime.

**Conclusion:**

Specific working conditions and certain personality traits are associated with variations in salivary cortisol concentrations. In addition, certain personality traits moderate the relationship between stressors and salivary cortisol concentrations. In conclusion, salivary cortisol concentrations at work seem to be modulated in part by personality traits.

## Background

Workplaces may be a major source of stress, and researchers need to understand where it originates and how employee personality traits influence the way workers adapt. The stress model assumes that exposure to environmental stressors (e.g., work, family, community) induces an endocrine (physiological) response to stress in the form of cortisol secretion. When cortisol secretion is dysregulated, it is associated with physical and mental health consequences. Salivary cortisol is particularly sensitive and reactive to environmental stressors [1–3], although it is not well known how personality traits could act to modify the relationship between workplace stressors and physiological stress responses.

This study examines how personality traits moderate the relationship between workplace organization conditions and cortisol secretion in a sample of 401 employees employed in 34 Canadian workplaces.

### Cortisol

Cortisol is a stress hormone and stressors provide the stimuli that trigger the secretion of cortisol [4]. The main components of the stress response are the hypothalamic-pituitary-adrenal (HPA) axis and the sympathetic part of the autonomic nervous system [5]. Increases in cortisol constitute a valid marker for the sustained activation of the HPA axis [6–8]. Cortisol secretions peak in early morning and decline throughout the remainder of the day [9].

Research has shown awakening cortisol to be a reliable measure of HPA axis activity because of its high intra-individual stability, which makes it suitable for measuring levels of daily stress and strain [5]. Cortisol mobilizes energy needed for different kinds of activities (sports, manual jobs, etc.) meant to promote adaptation to stressful environments [10]. Interestingly, low awakening cortisol levels have been associated with job burnout [11]. Even slight declines in cortisol levels during the day have shown associations with burnout [12–14]. In addition, high evening cortisol levels indicate a lack of adaptation among highly stressed individuals [5, 15, 16].

### The role of the workplace

#### Task design

*Decision latitude* is defined as the opportunity for employees to make work-related decisions and to influence their work group or company policies, or both [17]. Karasek [18] divides this concept into decision authority and skill utilization. *Decision authority* allows employees themselves to work out details such as how to organize their tasks and determine the pace at which to perform them [19]. *Skill utilization* refers to existing skills and qualifications that employees have as well as the potential for developing new ones. Some studies have found that control has a significant impact on salivary cortisol levels [3, 20, 21]. A study by Karlson et al. [12] concluded that having a low decision authority was significantly associated with higher cortisol secretion in the morning.

### Demands

*Physical demands* refer to workers exposure to health and safety risks, like high levels of noise, dust, vibration, etc. It also refers to physical efforts workers deployed carried out their job. Lower saliva cortisol levels have been observed in industrial workers on leisure days compared with work days [22].

*Psychological demands* have to do with the pace of work, the amount of work, and conflicting demands [18, 23]. Several studies seem to have concluded that psychological demands have no significant effect on cortisol secretion [5, 20, 24–31]. However, a study by Schlotz et al. [32], argued that overwork contributed to increases in awakening cortisol levels, while Karlson et al. [12] reported higher declining cortisol levels during day with excessive workload.

As for *number of hours worked and work schedule*, a study by Garde et al. [33] confirmed that differences in morning and afternoon cortisol concentrations were greater among employees who worked extended hours. A study by Marchand et al. [34] confirmed that number of hours worked acted as stressors in that they were positively associated with cortisol concentrations. Research by Lac and Chamoux [35] suggested that *irregular work schedules* led to increased levels of circadian cortisol.

### Social relations

*Social support* acts to acknowledge and support employees by making work more enjoyable and by compensating them for their efforts and for the challenges they must face in the workplace [36]. Some studies have shown that high levels of social support are associated with higher salivary cortisol levels [1, 37].

### Gratifications

Workplace gratifications are a major source of recognition, motivation validation, and security that encourage employees to invest themselves in their work. Low levels of gratification can cause dissatisfaction and stress that can affect employee mental health. The relationship between gratifications and salivary cortisol, to our knowledge, has yet to be established.

### The role of personality

Personality traits refers to the propensity to react in certain ways in given situations [38]. More specifically, the structure of personality traits is hierarchically organized, going from broad, general traits to narrower, more specific ones [39].

General traits are defined according to personality characteristics related to extraversion, agreeableness, neuroticism, conscientiousness and openness. *Extraversion* includes self-confidence, sociability, and the tendency to experience positive emotions such as joy and pleasure [40]. *Agreeableness* is seen as being naïve, kind, and cooperative individual [41]. Those with high scores on the *Conscientiousness* dimension are scrupulous, well-organized, motivated, hard-working, meticulous, persevering, and diligent [41]. *Neuroticism* refers to the tendency to experience negative emotions, nervousness, insecurity, social anxiety, and low self-esteem [42]. Finally, individuals with *Openness* are intellectually curious and have flexible outlooks [43], and have desire to learn lessons from experience [40].

Specific personality traits, like self-esteem and locus of control, apply to more explicit behaviors and may vary more than general traits from one situation to another. Rosenberg [44], for example, has defined self-esteem as the image that individuals create and hold of themselves and the approval or disapproval they feel as a result. Locus of control refers to the degree to which individuals feel that they exercise control over significant life events [45, 46]. Note that, to the best of our knowledge, no comprehensive study of the personality traits described above has been done to determine what direct impact, if any, they may have on salivary cortisol secretion on a worker population. The only exception has been a limited number of studies of self-esteem showing that it played no significant role in cortisol secretion [8, 21, 47].

### Theoretical model

The model we are proposing here incorporates biological, psychological, and social determinants of workplace stress derived from the model of Lazarus and Folkman [48], the social stress theory of Pearlin [49], and the multilevel model [50, 51]. When exposed to a stressor, the human organism will call on internal resources like adrenocortical response to combat it, and thereby avoid exhausting its resources. These reactions help individuals muster a rapid and effective coping response when faced with a threat or other demand [52]. Individuals who are exposed to the same stressor will not interpret the threat it poses in the same ways. As a consequence, personality traits (general and specific) are likely to moderate the impact that workplace stressors have on individual physiological responses to stress.

Some work organization conditions have shown statistically significant associations with cortisol levels. Decision latitude has been associated‚ directly and in a statistically significant way‚ with increases in salivary cortisol secretion at awakening and 30 min later [3, 12], and with late-evening cortisol levels (Sjogren et al. [21]. Next, psychological demands were also positively associated with awakening cortisol levels [12, 32, 53]. In addition, number of hours worked was positively associated with cortisol secretion [33, 34, 54]. Social support in the workplace was negatively associated with awakening cortisol secretion [1] and positively with evening cortisol levels [37]. The first hypothesis that emerges from those results is:

#### Hypothesis 1

Work organization conditions are associated to variations in salivary cortisol secretion.

According to Lazarus and Folkman [48], physiological stress responses to a threat depend on individual perceptions of the threat. Thus, various stressors that arise from work organization conditions are perceived as threats to a greater or lesser extent by the employees interpreting them. Pearlin [49], moreover, has reinforced this idea with findings that the same stressors do not have the same impact in different subjects. Marchand et al. [50, 51] agree with Pearlin, observing that whether structural components are considered constraints or resources depends on how actors interpret them, which is of crucial importance. In cases where personality traits attenuate perceptions of constraints or threats, the organism would also find that its need to prepare itself physiologically to fight was lessened. Conversely, if personality traits accentuate perceptions of constraints or threats, the organism will feel that much more compelled to activate the physiological mechanisms it needs to fight. Hence, we state a second hypothesis:

#### Hypothesis 2

The relationship between work organization conditions and salivary cortisol secretion is moderated by personality traits.

## Methods

### Participants

The data come from the SALVEO Study, which sought to highlight and differentiate the various factors affecting mental health problems. The data were collected between 2009 and 2012 from a sample of 34 Canadian employers randomly selected from a list of 500 companies insured by a large insurance company. For each employer, a random sample of employees was first selected to answer a questionnaire (*N* = 1301 employees, 66.7 % response rate). From these respondents a sample of 10 to 15 employees per institution was targeted to participate in the second phase of the research project, in which saliva samples were collected to evaluate cortisol levels within the same week or the week after they filled in the questionnaire. All told, 1043 employees were invited back, among whom 401 agreed to participate (39.9 % response rate) in the current sub-study (mean age for woman = 41.11 SD = 10.68) (mean age for men = 41.74 SD = 10.30). Women represented 56.1 % of the sample and had an average age of 41.3 years (standard deviation = 10.81). The research protocol received approval from the ethics committees of the University of Montreal, McGill University, Laval University, Bishops University, and Concordia University.

### Measures

#### Salivary cortisol

Consenting employees were asked to furnish 5 saliva samples per day (on awakening, 30 min after awakening, and at 2:00 p.m., 4:00 p.m., and bedtime), repeated three days each week (Saturday, Tuesday, and Thursday for most employees). The purpose was to enable measuring cortisol levels both in the workplace and away from the workplace. Participants were instructed not to eat large meals, smoke cigarettes, drink caffeinated beverages (e.g., tea, coffee, Coke), drink fruit juices, or consume dairy products (e.g., yogurt, milk, cheese). Moreover, they were asked to rinse their mouths with water so as to eliminate any traces of food deposits. They also were instructed not to brush their teeth, use dental floss, or take part in strenuous activity within two hours of sample collections.

The extent of compliance with these instructions was evaluated by having them maintain a log book in which they were to record the collection time for each sample. For sample respondents, 94.9 % reported that Saturday was their day off work; 5.1 % reported it as Tuesday or Thursday, which was carefully coded. Preliminary analyses established that participants had held to the sampling schedule and that potential effects from extraneous variables remained within expected ranges. For statistical reasons, the point of comparison was always set as the day off indicated in our baseline analyses. This was justified because it had been shown that cortisol concentrations rose between days off and work days [28, 34, 55], and it is considered the best focal point for determining the rhythmicity of diurnal cortisol profiles in any given work week.

The sampling times described above are generally reliable markers of the diurnal cortisol secretion cycle, as previous studies have shown [56–58]. To evaluate salivary cortisol levels, sputum collection tubes ("salivettes" from Sarstedt, Ville St-Laurent, Québec) were used. The procedure consists of inserting a straw into the mouth and expelling a small quantity of saliva into the tube. Participants were asked to keep the saliva samples in their refrigerator at home and to bring them to work with them when the weekly sample collection was complete. One week later a research assistant would come by to pick up the samples at the workplace. The samples were then immediately frozen and maintained at −20 °C until they were submitted for analysis. Salivary cortisol concentrations were determined in a laboratory at the Centre for Studies on Human Stress (CSHS) of the Institut universitaire en santé mentale de Montréal (IUSMM) using a radioimmunoassay kit from DSL (Diagnostic Systems Laboratories, Inc., Webster, Texas, USA), with minor modifications.

#### Workplace

*Skill utilization, decision authority and psychological demands and social support were* measured with the Job Content Questionnaire (JCQ) (1985) [59]. Responses were based on a 4-point Likert scale (strongly disagree-strongly agree). Skill utilization consisted of six items (Alpha = 0.80; e.g., my work requires me to learn new things), *Decision authority* contained 3 items (Alpha = 0.79; e.g., I have the freedom to decide how I do my work). *Psychological demands* were measured with nine items (Alpha = 0.73; e.g., my job requires working very fast). *Social support* from colleagues was measured with 4 items (Alpha = 0.83; e.g., the people I work with are helpful getting the job done). *Social support* from supervisors was measured with 4 items (Alpha = 0.89; e.g., my supervisor feels concerned about the well-being of subordinates). *Physical demands* and job insecurity were measured using the Effort-Reward Imbalance Questionnaire (1996) [60]. Responses were based on a 4-point Likert scale (strongly disagree-strongly agree). Physical demands were measured with one item (e.g., my work requires physical effort), and *job insecurity* with two items (Alpha = 0.65; e.g., I am experiencing or expect to experience an undesirable change in my work situation). *Number of hours* worked was obtained by summing each hour worked per week in all jobs. *Work schedule* was measured using a 4-point item (never/all the time) from the Québec Health and Social Survey (QHSS-98) (e.g., in your current job).

#### Personality traits

The Big Five personality traits were measured with the Mini International Personality Item Pool (Mini-IPIP) [61] using 20-item, 5-point scale (strongly disagree/strongly agree). *Openness* 4-item (Alpha = 0.68; e.g., I see myself as someone with a vivid imagination). *Conscientiousness* 4-item (Alpha = 0.63; e.g., I see myself as someone who gets chores done right away). *Extraversion* 4-item (Alpha = 0.78; e.g., I see myself as someone who is the life of the party). *Agreeableness* 4-item (Alpha = 0.70; e.g., I see myself as someone who sympathizes with others' feelings). *Neuroticism* 4-item (Alpha = 0.70; e.g., I see myself as someone who has frequent mood swings). *Self-esteem* was measured with the Rosenberg Self-Esteem Scale short version [44] using a 6-item, 5-point scale (strongly disagree/strongly agree) (Alpha = 0.87; e.g., you feel you have a number of good qualities). Locus of control was measured using a 7-item, 5-point scale developed by Pearlin and Schooler [62] (Alpha = 0.84; e.g., there’s nothing you can do to solve some of your problems).

#### Control variables

Previous studies have demonstrated the confounding effects that certain covariates have had on diurnal cortisol levels. We have, as a consequence, adjusted our statistical analyses to reflect the findings for the following covariates: self-reported time of awakening [63], sex [64], age [65], season of sampling [66], cigarette smoking [67], alcohol consumption [68], regular physical activity [69], psychotropic drug use [70], health problems [71], and body mass index [72]. Time of awakening was coded in hours and minutes. Sex was coded as 0 = male and 1 = female. Age was coded in years. Season of sampling (spring, summer, autumn, winter) measured with three dummy-coded indicators using spring as the reference category). Cigarette smoking was coded with a continuous variable showing the number of cigarettes smoked per day. For alcohol consumption respondents gave the number of alcoholic beverages consumed each day of the week. Physical activity over the preceding 3 months was measured by the frequency of physical activity lasting longer than 20 min. Respondents indicated frequency using a 7-point Likert-type scale (1 = never, 7 = 4 or more times per week). Medications prescribed during the preceding month were binary coded (1 = yes, 0 = no) for the use of at least one of the following medications: Valium, Ativan (tranquilizers); Prozac, Paxil, Effexor (antidepressants); aspirin, Tylenol, Motrin (analgesics); Imovane, Nytol, Starnoc (soporifics).

The variable for chronic physical health problems (i.e., those that lasted 6 months or longer and were diagnosed by a physician) reflected the presence of at least 1 of the following 29 conditions: food allergies, other allergies, asthma, fibromyalgia, arthritis or rheumatism, back pain, hypertension, migraines, chronic bronchitis, emphysema, chronic obstructive pulmonary disease, diabetes, epilepsy, heart disease, cancer, intestinal or stomach ulcers, cerebrovascular accidents (stroke), multiple sclerosis, urinary incontinence, inflammatory bowel diseases such as Crohn's disease, irritable bowel syndrome, cataract, glaucoma, thyroid disorders, chronic fatigue syndrome, multiple chemical sensitivity, schizophrenia, mood disorders (e.g., depression, bipolar disorder, manic disorder, dysthymia), anxiety disorders (e.g., phobia, obsessive-compulsive disorder, panic disorder).

Body mass index was derived by dividing weight in kilograms by height in meters squared. *Marital status* was coded as 0 = single, 1 = living as a couple, and *parental status* as 0 = no, 1 = yes. *Marital stress* was measured using a 4-item scale (yes/no) (Wheaton [73]) (Alpha = 0.70; e.g., your partner does not understand you). *Parental stress* was measured using a 3-item scale (yes/no) (Wheaton [73]) (Alpha = 0.60; e.g., one of your children seems very unhappy). *Educational level* was coded using the highest academic degree attained by the respondent on a 10-category scale which are rank ordered according to the number of years (lowest to highest) needed to complete each degree (1 = none, 2 = high school, 3 = professional school, 4 = college (general), 5 = college (technical), 6 = university (undergraduate certificate), 7 = university (bachelor’s degree), 8 = university (graduate diploma), 9 = university (master’s degree), 10 = university doctorate). *Household income* was coded using pre-tax household income for the preceding 12 months on a 12-category scale (1 = less than $20 000, 12 = $120 000 or more). *Social support outside the workplace* was derived using a 4-item scale (yes/no) (e.g., is there anyone in your circle of friends or family in whom you can confide and to whom you may speak freely about your problems?). Finally, the stressful childhood events (before age of 18) variable was measured using a 7-item, 2-point scale (yes/no) (Wheaton [73]) (e.g., are your parents divorced?).

### Statistical analyses

Multilevel regression models [74–77] were used to assess cortisol concentrations at the following levels: sampling days (Level 1) nested in employees (Level 2), employees nested in companies (Level 3). This statistical approach allowed considering the data as a whole when estimating cortisol variations between levels. The model included three time of the day dummy-coded variables (awakening is the reference category) indicating cortisol samples at occasion-2 (30 min after awakening), at occasion-3 (2:00 PM), at occasion-4 (4:00 PM), and at occasion-5 (bedtime). Next, two binary variables indexed cortisol concentrations on Work Day 1 and Work Day 2, leaving day off as the reference category. Our analysis strategy involved entering in a variance component model‚ workplace, personality, and control variables so that their main effects could be evaluated. Analyses were carried out separately for specific traits and general traits to avoid potential collinearity yielded by correlated specific and general traits.

To test interactions, each interaction, including main effects, between work and personality variables were estimated separately, and all significant interactions were then re-estimated in one model. Model parameters were estimated by the restricted iterative generalized least-squares (RIGLS) method, of MlwiN 2.26 software [78]. To reduce the asymmetrical distribution and improve the convergence of the estimation algorithm, cortisol concentrations in ug/dl were multiplied by 100 and log transformed (natural logarithm). The significance of the combined contribution of the variables and of each individual regression coefficient was evaluated using a two-tailed probability for rejection of the null hypothesis set at *p* ≤ 0.05. Random coefficients were examined using halved p values [74].

## Results

Table 1 presents the descriptive statistics for the sample.

**Table 1:** Descriptive statistics

	Min–Max	Mean/proportion	Standard deviation
STRESS RESPONSE			
Cortisol	0.00–4.07	0.18	0.22
WORK			
Skill utilization	6–24	18.27	3.04
Decision authority	3–12	8.81	1.84
Psychological demands	13–36	23.27	3.77
Physical demands	1–4	1.77	0.85
Number of hours worked	17–65	39.22	5.10
Work schedule (irregular)	1–4	1.45	0.65
Social support from coworkers	6–16	12.76	1.93
Social support from supervisor	4–16	12.45	2.42
Job insecurity	2–8	3.77	1.28
PERSONALITY			
Self–esteem	9–24	19.80	3.12
Locus of control	3–28	20.00	4.20
Extraversion	4–20	12.98	3.31
Agreeableness	4–20	15.91	2.44
Neuroticism	4–18	10.28	2.80
Conscientiousness	4–20	15.18	2.48
Openness	7–20	14.48	2.78
CONTROL VARIABLES			
Sex (female)	0–1	0.55	
Age	19–65	41.40	10.51
Education level	1–10	5.20	2.16
Household income	1–12	7.50	3.26
Social support outside workplace	0–1	0.82	
Stressful life events (childhood)	0–6	1.09	1.22
Marital status (living as couple)	0–1	0.74	
Parental status (present)	0–4	0.89	1.04
Marital stress	0–4	0.48	0.88
Parental stress	0–3	0.21	0.56
Tobacco use	0–25	1.09	3.82
Body mass index	17.13–68.25	29.86	6.94
Alcohol consumption	0–42	4.45	5.44
Psychotropic drug use	0–1	0.10	
Chronic health problems	0–5	1.03	1.23
Physical activity	1–7	4.31	2.00
Season–Winter		0.21	
Season–Spring		0.46	
Season–Summer		0.11	
Season–Autumn		0.23	
Awakening time	2–12.47	6.86	1.51

Preliminary analyses evaluated how well participants adhered to the protocol for saliva collection, which took place at 30-min intervals [14]. Such evaluations, however, could not be performed for the samples collected at awakening and at bedtime. The proportion of participants who complied with the saliva collection protocols at approximately 30-min intervals was 98.5 % (30 min after awakening), 72.6 % (at 2 p.m.), and 64.8 % (at 4 p.m.). Calculating overall compliance with the protocol revealed that 60.9 % of participants followed the protocol fully. In the final analysis, the results of our multilevel regression analyses did not change in any statistically significant way after adding total compliance as a control variable. We therefore removed compliance from our later analyses. Table 2 presents the results of main effects that workplace conditions and specific personality traits had on cortisol concentrations.

**Table 2:** Main effects of work and specific personality traits on cortisol concentrations (unstandardized coefficients)

	At awakening	After 30 min	2 p.m.	4 p.m.	At bedtime
Fixed part					
Constant (Day off)	2.882**	3.107**	1.982**	1.566**	1.004**
Workday 1	0.137**	0.354**	−0.016	−0.037	0.039
Workday 2	0.185**	0.387**	−0.020	−0.088*	0.074
WORK					
Skill utilization	−0.014	−0.009	−0.011	−0.009	0.015
Decision authority	0.018	−0.000	0.015	−0.006	−0.025
Psychological demands	−0.006	−0.002	−0.018	0.001	−0.034**
Physical demands	−0.043	−0.041	−0.022	0.028	0.015
Number of hours worked	−0.002	−0.002	0.010	0.001	0.001
Work schedule (irregular)	0.046	0.033	0.076	0.049	0.239
Support from coworkers	−0.022	−0.013	−0.008	−0.031	0.002
Support from supervisors	0.006	0.014	−0.007	0.012	−0.017
Job insecurity	−0.021	−0.021	−0.034	−0.059*	−0.077*
PERSONALITY					
Self-esteem	−0.016	−0.011	−0.010	0.006	−0.016
Locus of control	−0.005	0.000	0.005	−0.008	0.000
Random part and fit					
σ^2^ (companies)	0.021**	0.026**	0.022	0.018	0.026
σ^2^ (employees)	0.107**	0.129**	0.146**	0.253**	0.354**
σ^2^ (samples)	0.220**	0.249**	0.258**	0.316**	0.419**
*χ*2	12858.4**	12724.7**	12731.4**	12452.4**	12174.7**
df	33	33	33	33	33

Note A: *p ≤ 0.05 and **p ≤ 0.01

Note B: The following variables were controlled for in all models: gender, age, educational level, household income, social support outside the workplace, stressful childhood events, marital status, parental status, marital stress, parental stress, smoking, BMI, alcohol, psychotropic drugs, chronic health problems, physical activity, season, time of awakening

The first model shows a significant difference between the cortisol secreted on work days and day off at awakening and 30 min later. Employees secreted more cortisol in the morning before going to work than on their day off. Also, psychological demands are associated with lower salivary cortisol levels at bedtime. In addition, job insecurity was negatively associated with cortisol secretion at 4 p.m. and at bedtime. The more pronounced the experience of job insecurity, the lower the concentration of salivary cortisol at 4 p.m. and at bedtime. Moreover, no specific personality trait had a significant impact on cortisol concentrations.

Table 3 presents the results of main effects of work and general personality traits on cortisol concentrations.

**Table 3:** Main effects of work and general personality traits on cortisol concentrations (unstandardized coefficients)

	At awakening	After 30 min	2 p.m.	4 p.m.	At bedtime
Fixed part					
Constant (Day off)	2.904**	3.108**	1.940**	1.574**	1.015**
Workday 1	0.136**	0.354**	−0.016	−0.038	0.040
Workday 2	0.185**	0.387**	−0.019	−0.088*	0.073
WORK					
Skill utilization	−0.016	−0.013	−0.014	−0.007	0.016
Decision authority	0.015	0.003	0.023	−0.009	−0.027
Psychological demands	−0.005	0.001	−0.015	−0.000	−0.035**
Physical demands	−0.037	−0.041	−0.017	0.025	0.018
Number of hours worked	−0.003	−0.003	0.010	0.001	0.001
Work schedule (irregular)	0.050	0.024	0.072	0.040	0.231
Support from coworkers	−0.025	−0.017	−0.004	−0.026	0.000
Support from supervisors	0.007	0.013	−0.009	0.011	−0.016
Job insecurity	−0.026	−0.020	−0.040	−0.061*	−0.080*
PERSONALITY					
Extraversion	0.012	0.002	0.003	0.013	0.009
Agreeableness	−0.028*	−0.015	−0.031*	−0.034	−0.019
Neuroticism	0.018	−0.010	−0.009	0.011	0.016
Conscientiousness	−0.001	0.006	−0.007	0.012	0.009
Openness	0.007	−0.000	−0.000	0.005	−0.011
Random part and fit					
σ^2^ (companies)	0.023**	0.028**	0.025**	0.021	0.026
σ^2^ (employees)	0.106**	0.129**	0.145**	0.251**	0.357**
σ^2^ (samples)	0.220**	0.250**	0.258**	0.316**	0.419**
*χ*2	12862.2**	12725.0**	12735.9**	12456.4**	12176.1**
df	36	36	36	36	36

Note A: *p ≤ 0.05 and **p ≤ 0.01

Note B: The following variables were controlled for in all models: gender, age, educational level, household income, social support outside the workplace, stressful childhood events, marital status, parental status, marital stress, parental stress, smoking, BMI, alcohol, psychotropic drugs, chronic health problems, physical activity, season, time of awakening

The results of these analyses revealed a significant difference between cortisol concentrations at awakening and 30 min later on work days and day off. Cortisol concentrations were higher on work day mornings than on the mornings of day off. Moreover, psychological demands were negatively associated with cortisol concentrations at bedtime, that is, the heavier the psychological demands, the lower the salivary cortisol concentrations at bedtime. Also, job insecurity was similarly associated with cortisol concentrations at 4 p.m. and at bedtime. That is, the greater the job insecurity, the lower the salivary cortisol concentrations at bedtime. Agreeableness was also associated with lower cortisol concentrations at awakening and at 2 p.m.

All in all, the results of Tables 2 and 3 show that cortisol concentrations varied significantly over time, between individuals and between employers, once they were adjusted for all variables. The one exception was the absence of significant variations between employers for cortisol levels at 4 p.m. and at bedtime.

**Fig. 1:**
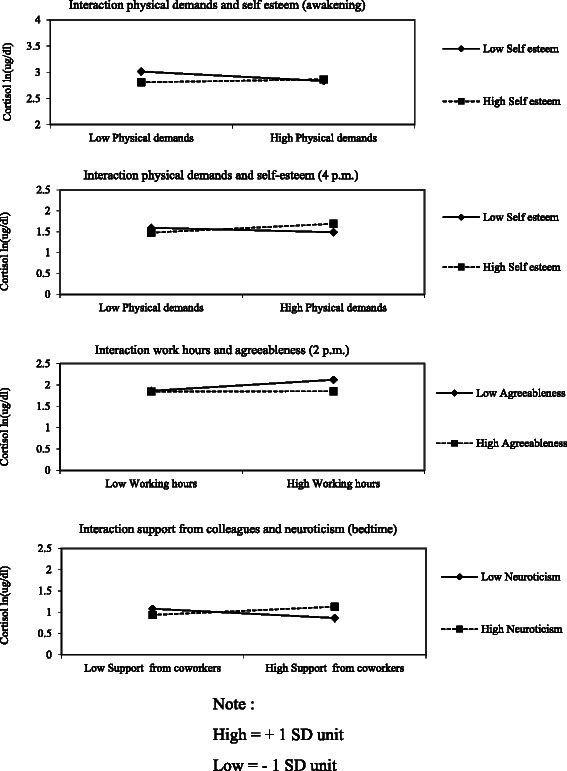
Interactions between personality and work organization conditions. High = + 1 SD unit. Low = − 1 SD unit

Finally, we evaluated the interactions, including main effects, between work organization conditions and personality traits. In the case of specific personality traits on awakening cortisol, we tested two interactions that were previously significant when tested separately and one of the two remained significant (self-esteem and physical demands/ self-esteem and coworker support). Results gave *χ*^2^ = 6.907, df = 2, *p* = .032, but only self-esteem and physical demands interaction was significant (b = 0.022, *p* = 0.020). Also, we tested three interactions that were previously significant when tested separately on cortisol at 4 p.m. (self-esteem and psychological demands/ self-esteem and physical demands/ locus of control and psychological demands). Combined testing gave *χ*^2^ = 8.516, df = 3, *p* = .036, and only self-esteem and physical demands interaction was significant (b = 0.030, *p* = 0.024). For general traits, we tested two interactions that were previously significant when tested on cortisol at 2 p.m. (agreeableness and working hours/ conscientiousness and skill utilization). We obtained *χ*^2^ = 7.159, df = 2, *p* = .028 (b = −0.005, *p* = 0.020), and only agreeableness moderated the relationship between high working hours and cortisol at 2 p.m. Additionally two interactions were tested that were previously significant when tested separately on cortisol at bedtime (neuroticism and coworker support/ consciousness and job insecurity). Results gave (*χ*^2^ = 7.4656, df = 2, *p* = .024), and only neuroticism interacted with coworker support for cortisol at bedtime (b = 0.019, *p* = 0.011). Table 4 reports the regression coefficients of significant interactions, and Fig. 1 illustrates these interactions graphically.

**Table 4:** Significant interactions on cortisol concentrations (unstandardized coefficients)

	Awakening	2 p.m.	4 p.m.	Bedtime
1) Physical demands by self-esteem				
Constant	2.885**			
Physical demands	−0.037			
Self-esteem	−0.014			
Interaction	0.022*			
2) Physical demands by self-esteem				
Constant			1.561**	
Physical demands			0.031	
Self-esteem			0.007	
Interaction			0.030*	
3) Work hours by Agreeableness				
Constant		1.912**		
Work hours		0.013*		
Agreeableness		−0.029*		
Interaction		−0.005*		
4) Support form colleagues by neuroticism				
Constant				1.002**
Support from colleagues				−0.003
Neuroticism				0.011
Interaction				0.019*

Note A: *p ≤ 0.05 and **p ≤ 0.01

Note B: The following variables were controlled for in all models: skill utilization, decision authority, psychological demands, physical demands, number of hours worked, work schedule, support from coworkers, support from supervisors, locus of control (1 and 2), extraversion (3 and 4), conscientiousness (3 and 4), openness (3 and 4), gender, age, educational level, household income, social support outside the workplace, stressful childhood events, marital status, parental status, marital stress, parental stress, smoking, BMI, alcohol, psychotropic drugs, chronic health problems, physical activity, season, time of awakening

Cortisol concentrations at awakening are higher for employees with low physical demands and low self-esteem. Second, cortisol concentrations at 4 p.m. are higher for employees with high physical demands and high self-esteem. Third, high working hours is associated with higher cortisol concentrations at 2 p.m. when agreeableness is low. When agreeableness is high, cortisol concentrations at 2 p.m. are lower but do not appear to be related to work hours. Finally, cortisol concentrations at bedtime were higher for employees with high coworker support and high neuroticism.

## Discussion

The objective of this study was to evaluate whether personality traits explain the relationship between workplace stressors and the cortisol concentration. Study results indicated that a significant difference in salivary cortisol concentrations existed between levels found on work days and those on days off [34]. All model estimations supported this observation, primarily for morning cortisol levels. More specifically, we found that cortisol levels at awakening and 30 min later were significantly higher on work days. These results concord with those of a number of other studies [1, 13, 14, 26, 28, 34, 55, 79]. Employees are, in effect, physiologically preparing themselves for potentially stressful situations that may arise during work days.

Our study provides partial support for Hypothesis 1 (H1), which posits that working conditions contribute directly to salivary cortisol secretions. Psychological demands were in fact associated with a lower salivary cortisol level at bedtime. Job insecurity was also associated with lower cortisol concentrations at 4 p.m. and at bedtime. The associations for both psychological demands and job insecurity suggest the presence of mental health markers, since these results support those obtained by Marchand et al. [13]. That study maintained that, compared to low symptoms subjects, people suffering from psychological distress, burnout, and depression had lower cortisol levels during the day compared to low symptoms subjects.

Hypothesis 2 (H2), which posits that personality traits have a moderating effect on the relationship between work organization conditions and salivary cortisol concentrations, was partially supported by the results of our study. Self-esteem interacted significantly with the relationship between physical demands and cortisol levels at awakening and at 4 p.m. Cortisol levels at awakening were higher for employees with low physical demands and low self-esteem. Also, cortisol concentrations at 4 p.m. were higher for employees with high physical demands and high self-esteem. Self-esteem thus moderated the impact of physical demands on cortisol levels, and seemed to act as a protective factor. Cortisol levels tend to fall more during the day for individuals with certain mental health problems (e.g., psychological distress, burnout, depression) [13]. This leads us to conclude that higher self-esteem makes it possible to reverse this decline when physical demands are high.

Agreeableness interacted significantly with the relationship between number of hours worked and cortisol levels at 2 p.m. High working hours was associated with higher cortisol concentrations at 2 p.m. when agreeableness is low. When agreeableness is high, cortisol concentrations at 2 p.m. were lower but do not appear to be related to work hours. Given that cortisol levels tend to fall more sharply throughout the day for employees with burnout [12], it is arguable that agreeableness limits, but does not reverse, this decline.

Likewise, neuroticism interacted with the relationship between coworker support and cortisol levels at bedtime. Cortisol concentrations at bedtime were higher for employees with high coworker support and high neuroticism. Clearly, then, neuroticism can be said to modify the relationship between stressor and stress response.

Overall, we have found that higher self-esteem, agreeableness, and lower neuroticism personality traits play moderating effects on the relationship between cortisol secretions and some work stressors. These personality traits might thus facilitate adapting to stressors by reducing subsequent stress responses. These results are coherent with our theorization which states that some individual characteristics may act as moderators that influence individual interpretation. Since perceptions vary according to personality traits, they are apt to exacerbate or attenuate individually experienced effects and perceptions of constraints. One might expect that the explanation for these results has to do with the favorable self-image that individuals with high self-esteem have, which better disposes them to cope with stressors. This conclusion concord with the experimental study on the general population by Pruessner et al. [80] which states that subjects scoring high in self-esteem might have been able to interpret situations as unrelated to their general ability to perform in demanding situations, and thus did not interpret the test situation as threatening.

Agreeableness is characterized by altruism, kindheartedness, and naïveté, leading us to suppose that agreeable people are more inclined to deal positively with stressors. Neuroticism, by contrast, implies experiencing negative emotions, anxiety, and powerlessness. In addition, neuroticism is associated with the use of ineffective adaptation strategies. These outcomes are thus not surprising to find since, when individuals with high neuroticism encounter the stressors of daily life, rather than deploying positive and effective strategies for adapting, they react with negative thoughts [42].

This study has certain limitations. First, secondary data from the SALVEO Study restricted our choice of both measures and variables. Second, the selection of study participants by recruiting volunteers and the low response rate caused a selection bias. Third, the sample we used in this study was heterogeneous for a number of factors known to affect cortisol levels, particularly medications and health conditions. Even if strict exclusion criteria are normally applied when biological mechanisms are under study, doing so would likely have limited the generalizability of our results, which emerged after using a defined set of control variables. Fourth, the lack of consistency among studies evaluating cortisol levels may be due in part to the fact that employee samples were often homogeneously specific to one occupation (e.g., nurses, social workers). Fifth, sleep duration has been shown to associate with morning cortisol [10, 81] and will need to be controlled for in further study. However, the present study controlled for time of awakening in order to account for cortisol variation related to varying individual awakening time.

These employees likely experienced workplace stressors typical of their occupations. Moreover, we found that employers, according to our multilevel regression analyses, were a significant source of variation in cortisol levels. Further studies will be needed to evaluate how companies’ characteristics (e.g., firm size, organizational culture, economic sectors) may explain these variations. Fifth, when measuring cortisol levels, it would have been preferable for indicators of protocol compliance to have been measured with an electronic monitoring. Although participants did maintain logs for noting the times samples were taken, research using electronic measuring technology has revealed that participants are less accurate in their record-keeping than they should be [82]. This inaccuracy is likely to have occasioned variations in data collection times among participants. Variations caused by protocol noncompliance are difficult to evaluate, but compliance in this study showed no significant differences [14]. Therefore, the extent of protocol compliance did not significantly influence the results.

## Conclusions

In summary, this study has shown that several personality traits moderate the association between some work organization conditions and cortisol concentrations. Workplace interventions that target work organization conditions associated with cortisol levels that reach their lowest levels in the evening, particularly psychological demands and job insecurity, could prove worthwhile, especially since these two conditions do not appear to be moderated by personality traits. However, self-esteem, agreeableness, and neuroticism are personality traits that interact in ways that affect the relationship between physical demands, work hours and social support from colleagues on cortisol levels. Unlike the other two traits, self-esteem is a specific personality trait that may change over the course of a lifetime as the result of particular experiences or circumstances. Specific personality traits are actually the traits most susceptible to being changed by exogenous influences. Accordingly, it would be possible to intervene with training programs designed to raise self-esteem, such as workplace-based coaching by industrial psychologists. In addition, human resources management services might prove useful for enhancing career management and advancement. Psychometric testing could help ensure that when employees are approached about being promoted to demanding high-level positions, they have sufficiently high levels of self-esteem. Organizations could prepare potential candidates for promotion by building their self-esteem to levels appropriate to their new responsibilities. Ensuring good fits between employees and positions could also reduce the likelihood of undesirable outcomes for both employees and organizations. The same rationale could apply to hiring and staffing activities.

Although this study does discuss factors affecting variations in cortisol concentrations and the moderating role of personality traits, a number of elements remain to be clarified in future research. It would be particularly interesting, for example, to confirm the mediating effect of cortisol levels on the relationship between work organization conditions and burnout to see whether and how different cortisol patterns are associated with mental health. Future research might also consider including coping strategies to explain cortisol variations, as coping strategies relate to personality. Conducting further research into the effects, both direct and moderating, of personality traits on cortisol levels would also be essential for expanding and reinforcing our still somewhat limited understanding of the subject.
